# Late surgical start time is associated with increased blood transfusion following gastric bypass surgery

**DOI:** 10.1371/journal.pone.0282139

**Published:** 2023-02-24

**Authors:** Ziyad O. Knio, Lena Zhang, David A. Watts, Zhiyi Zuo

**Affiliations:** 1 Department of Anesthesiology, University of Virginia Health, Charlottesville, VA, United States of America; 2 School of Medicine, University of Virginia, Charlottesville, VA, United States of America; Morgagni-Pierantoni Hospital, ITALY

## Abstract

**Background:**

Surgical start time (SST) has demonstrated conflicting effects on perioperative outcomes due to confounding factors, such as increased acuity in later SST cases. This study investigated the effect of SST on blood transfusion after gastric bypass surgery, a complication-prone elective surgical procedure.

**Methods:**

This retrospective cohort study included all patients undergoing gastric bypass surgery at a single academic medical center from 2016 through 2021 (n = 299). The primary independent variable was SST (before vs. after 15:00). The primary outcome was blood transfusion. Secondary outcomes included postoperative respiratory failure, length of stay, acute kidney injury, and mortality. The associations between SST and outcomes were investigated with univariate analyses. Multivariate and receiver operating characteristic (ROC) analyses were applied to the primary outcome, adjusting for demographic and operative characteristics.

**Results:**

On univariate analysis, 15:00–18:43 SST was associated with an increased risk of blood transfusion (relative risk 4.32, 95% confidence interval 1.27 to 14.63, p = 0.032), but not postoperative respiratory failure, acute kidney injury, length of stay, or mortality. On multivariate analysis, the only independent predictor of postoperative blood transfusion was a 15:00–18:43 SST (adjusted odds ratio 4.32, 95% confidence interval 1.06 to 15.96, c-statistic = 0.638). ROC analysis demonstrated that compared to the 15:00 threshold, a 14:34 threshold predicted postoperative blood transfusion with better accuracy (sensitivity = 70.0%, specificity = 83.0%).

**Conclusions:**

Despite having similar demographic and operative characteristics, gastric bypass patients in the late SST cohort had a greater incidence of postoperative blood transfusion in this single-center study.

## 1. Introduction

Temporal trends in morbidity and mortality are increasingly garnering attention, as in-hospital morbidity and mortality are observed in greater frequency on weekends and at night [1, 2]. These trends are often attributed to provider-related factors, such as provider fatigue and inadequate staffing [3]. This should come as no surprise, as the effect of sleep on psychomotor performance and decision-making has been studied extensively [4, 5].

The effect of surgical start time (SST) on postoperative complications is an ongoing focus of research. It has been studied in many specialties, often with mixed results [6–25]. A significant association, when identified, often points towards unfavorable outcomes for groups with later SST [6–12]. The potential association between complications and SST is confounded by several factors, as case urgency and patient comorbidity profile are often increased in late SST cohorts [13–17]. It is also difficult to quantify the degree to which operating conditions vary with SST, especially across institutions [17]. A study team has hypothesized that clinical response to an intervention is significantly impacted by circadian variations at the cellular level [8]. Regardless, the effect of SST, if any, is important as it represents a potentially modifiable risk factor.

Gastric bypass surgery results may offer significant insight into the association between SST and postoperative complications, as this surgery represents a truly elective, non-urgent surgery with modifiable SST. Additionally, operative duration is relatively uniform and patients are well-optimized prior to surgery, yet postoperative complications are still observed in modest frequency [26, 27]. Specifically, blood transfusion occurs in 1.6% patients with gastric bypass surgery and 6.2% patients with revision surgery [28]. Blood transfusion is associated with significant side-effects and complications [28, 29]. Also, blood is a precious medical resource [30]. Thus, we designed this study to investigate whether SST is associated with the incidence of postoperative blood transfusion in the gastric bypass population at a single institution.

## 2. Materials and methods

### 2.1 Patient population, ethics approval

This retrospective cohort study investigated all gastric bypass surgeries performed at a single academic medical center (University of Virginia Health) from June 2016 through June 2021. All data were retrieved from the institutional electronic medical record, with queried variables collected *a priori* as part of routine care.

The study protocol (IRB-HSR #23603) was approved by the Institutional Review Board with waiver of written consent (approval date: January 4, 2022).

### 2.2 Inclusion and exclusion criteria

After the initial data query, the following criteria were applied. Gastric bypass surgery was defined by the following standardized Current Procedural Terminology (CPT) codes: 43644, 43645 (open gastric bypass); 43846, 43847 (laparoscopic gastric bypass); 43644, S2900 (robotic gastric bypass). All cases with an emergency case designation were excluded. Similarly, revision cases were excluded from analysis because the revision surgery may be very different in the complexity and urgency, and the conditions of patients may be variable. An independent reviewer identified that greater than 95% of the gastric bypass case volume was performed by just two surgeons during the five-year study period. Accordingly, cases performed by other surgeons were excluded from the primary analysis.

### 2.3 Measurements and data handling

The independent variable of interest was SST, denoted by “case start” in the anesthetic record. SST was discretized into two cohorts: before versus after 15:00. For generalizability, surgeons are allowed to book a 10-hour block per day and the institutional operations at this academic medical center are optimized for elective cases concluding before 17:00. The institution-specific operating room staffing change time of 15:00 served as the rationale for this discretization scheme. A 15:00 threshold has been used in existing studies performed at institutions with similar resources and practices [7, 9]. Case volume by SST was graphically visualized prior to any subsequent analysis in order to subjectively assess validity.

Independent variables also included age, sex, body mass index (BMI), American Society of Anesthesiologists (ASA) physical status classification, diabetes diagnosis, hypertension diagnosis, preoperative blood hemoglobin concentrations, preoperative blood creatinine concentrations, surgeon, operative time, mean expired sevoflurane concentration, anesthetic technique (volatile versus volatile-sparing, defined as a mean expired sevoflurane concentration of < 1.0%), and estimated blood loss.

### 2.4 Primary and secondary outcomes

The primary outcome was the need for postoperative blood transfusion within thirty days of index surgery, for several reasons. Importantly, bleeding and transfusion are risk factors for surgical complications [28, 29]. Estimated blood loss was initially considered as a potential primary outcome, however there exists considerable subjective variability in the documentation of this parameter. Rather, current blood banking practices have helped to ensure that transfusions are reliably captured in all phases of patient care. In other words, blood product administration was not subject to error by clinician documentation or billing practices. Finally, it was anticipated that the incidence of major adverse outcome events would be low. Postoperative blood transfusion events were further investigated by querying first postoperative hemoglobin concentration, nadir postoperative hemoglobin concentration, greatest 48-hour reduction in postoperative hemoglobin concentration, and discharge hemoglobin concentration. Time to transfusion and relevant intraoperative and postoperative events were also queried.

Secondary outcomes included postoperative respiratory failure, postoperative sepsis, postoperative venous thromboembolism, myocardial infarction within thirty days, cerebrovascular accident within thirty days, acute kidney injury, hospital length of stay, and mortality.

Postoperative respiratory failure, postoperative sepsis, postoperative venous thromboembolism, hospital length of stay, and mortality are discrete elements in the institutional electronic medical record. Myocardial infarction and cerebrovascular accident were defined by their International Classification of Disease (ICD)-10 codes (I21.0-I21.9 and I63.0-I63.9, respectively). Acute kidney injury was defined according to the Kidney Disease: Improving Global Outcomes criteria, applied to preoperative and postoperative serum creatinine recordings [31].

### 2.5 Sample size calculation

An *a priori* power analysis was conducted. In order to detect a moderate effect size (ω = 0.3) between the two SST cohorts with 80% power, a sample size of n > 88 was needed [32]. The final sample size obtained by applying the above inclusion and exclusion criteria to this observational study of consecutive cases acceptably exceeded this minimum value.

### 2.6 Statistical analysis

Statistical analysis was performed with R version 4.2.0 (R Core Team, Vienna, Austria) [33]. Continuous variables were summarized by mean ± standard deviation, while categorical variables were summarized by frequency (%). All hypothesis tests were two-sided, with significance defined by α ≤ 0.05.

Baseline differences between SST cohorts were investigated with univariate analyses. Student’s t-test was applied to continuous variables while Fisher’s exact test was applied to categorical variables. Associations between SST and the primary and secondary outcomes were also investigated with the above univariate tests.

A multivariate analysis was then conducted in order to adjust for potential confounders of postoperative blood transfusion and/or SST. Preoperative and intraoperative variables demonstrating significant association (p < 0.05) with postoperative blood transfusion on univariate analysis were considered potential predictors in the multiple logistic regression model. Variable selection was accomplished by backwards stepwise model adjustment by Akaike information criterion. Adjusted odds ratio (AOR) and accompanying confidence interval are reported for the independent predictor(s) of blood transfusion. Model discrimination was assessed with the c-statistic [34].

A manual audit was performed, comparing first postoperative hemoglobin concentration, nadir postoperative hemoglobin concentration, greatest 48-hour reduction in postoperative hemoglobin concentration, and discharge hemoglobin concentration between the two SST cohorts and between the transfused and non-transfused cohorts. Student’s t-test was applied. Intraoperative and postoperative events in the transfused cohort were also queried.

A *post-hoc* receiver operating characteristic (ROC) analysis was applied to investigate whether the sensitivity and specificity for SST predicting postoperative blood transfusion could be improved under a different discretization scheme [35].

## 3. Results

### 3.1 Surgical start time distribution

**Fig 1:**
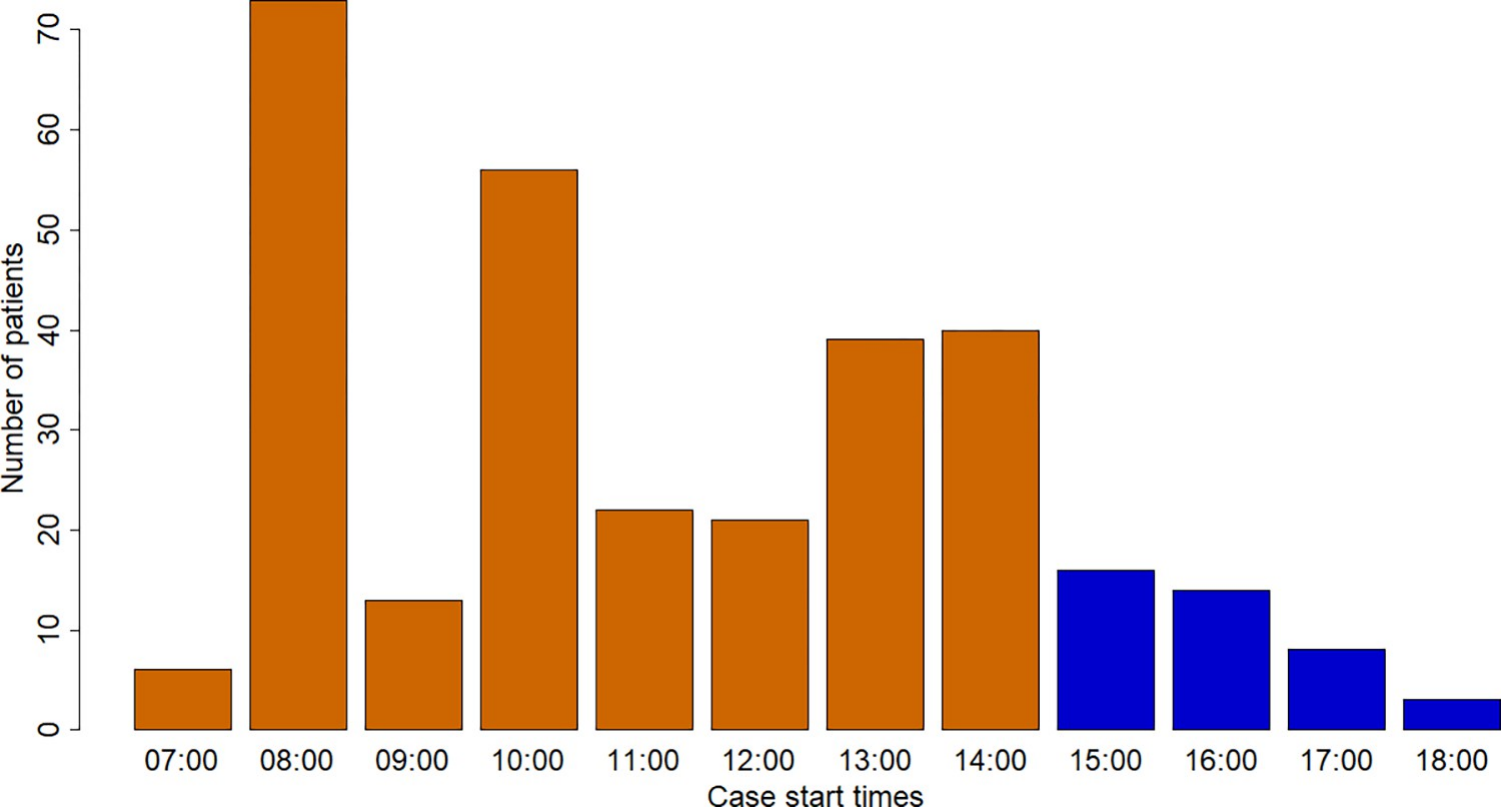
Distribution of surgical case start times.

Of the 311 patients who underwent elective index gastric bypass surgery at a single academic medical center from June 2016 to June 2021, there were 299 patients meeting inclusion criteria. The aggregate range of SSTs was 07:53–18:43. The distribution of SSTs is provided in Fig 1. There were 259 patients in the 07:53–14:59 SST cohort and 40 patients in the 15:00–18:43 SST cohort.

### 3.2 Patient data

The average age was 46.76 ± 11.23 years, male sex comprised 14.0% of patients, and the average BMI was 48.33 ± 10.64 kg/m^2^. Patients had an ASA physical status of ASA 2, ASA 3, or ASA 4. There were no significant differences in these baseline characteristics when comparing the 07:53–14:59 SST and 15:00–18:43 SST cohorts. Specifically, medical comorbidities (diabetes, hypertension) and baseline laboratory values (creatinine, hemoglobin) were comparable between the two SST cohorts. Distribution of surgeon, distribution of surgical approaches (open, laparoscopic, robotic), operative time, volatile anesthetic use, and estimated blood loss were all found to be similar between the 07:53–14:59 SST and 15:00–18:43 SST cohorts (Table 1).

**Table 1:** Demographic data and univariate comparisons between SST cohorts.

Characteristic	Aggregate n = 299	07:53–14:59 SST n = 259	15:00–18:43 SST n = 40	n	p-value	Difference or RR [95% CI]
Age (years)	46.76 ± 11.23	46.90 ± 11.39	45.85 ± 10.26	299	0.556	-1.05 [-4.60 to 2.50]
Sex (male)	42/299 (14.0%)	34/259 (13.1%)	8/40 (20.0%)	299	0.231	1.52 [0.76 to 3.05]
BMI (kg/m^2^)	48.33 ± 10.64	48.37 ± 10.54	48.08 ± 11.40	299	0.878	-0.30 [-4.15 to 3.55]
ASA 2	112/299 (37.5%)	97/259 (37.5%)	15/40 (37.5%)	299	1.000	
ASA 3	183/299 (61.2%)	158/259 (61.0%)	25/40 (62.5%)	299	1.000	
ASA 4	4/299 (1.3%)	4/259 (1.5%)	0/40 (0.0%)	299	1.000	
Diabetes	153/299 (51.2%)	132/259 (51.0%)	21/40 (52.5%)	299	0.867	1.03 [0.75 to 1.42]
Hypertension	163/299 (54.5%)	137/259 (52.9%)	26/40 (65.0%)	299	0.174	1.23 [0.95 to 1.59]
Preoperative Hemoglobin (g/dL)	13.20 ± 1.45	13.15 ± 1.42	13.46 ± 1.60	190	0.357	0.31 [-0.36 to 0.97]
Preoperative Creatinine (mg/dL)	0.88 ± 0.59	0.89 ± 0.62	0.86 ± 0.29	194	0.748	-0.02 [-0.17 to 0.12]
15:00–18:43 SST	40/299 (13.4%)	-	-	299	-	-
Weekend	18/299 (6.0%)	1/259 (0.4%)	17/40 (42.5%)	299	<0.001	110.07 [15.06 to 804.54]
Surgeon A	150/299 (50.2%)	129/259 (49.8%)	21/40 (52.5%)	299	0.865	1.05 [0.77 to 1.45]
Surgeon B	149/299 (49.8%)	130/259 (50.2%)	19/40 (47.5%)	299	0.865	0.95 [0.67 to 1.34]
Bypass—Open	15/299 (5.0%)	12/259 (4.6%)	3/40 (7.5%)	299	0.255	
Bypass—Laparoscopic	224/299 (74.9%)	198/259 (76.4%)	26/40 (65.0%)	299	0.255	
Bypass—Robotic	60/299 (20.1%)	49/259 (18.9%)	11/40 (27.5%)	299	0.255	
Concurrent Procedure	82/299 (27.4%)	73/259 (28.2%)	9/40 (22.5%)	299	0.569	0.80 [0.44 to 1.46]
Operative Time (min)	171.63 ± 44.09	170.70 ± 44.65	177.60 ± 40.25	299	0.325	6.90 [-7.02 to 20.81]
Mean Expired Sevoflurane (%)	1.62 ± 0.49	1.61 ± 0.50	1.65 ± 0.44	257	0.689	0.03 [-0.13 to 0.19]
Mean Expired Sevoflurane <1.0%	27/257 (10.5%)	24/220 (10.9%)	3/37 (8.1%)	257	0.777	0.74 [0.24 to 2.34]
Estimated Blood Loss (mL)	42.53 ± 60.85	41.84 ± 61.83	48.53 ± 52.76	165	0.632	6.69 [-21.91 to 35.29]
Postoperative Blood Transfusion	10/299 (3.3%)	6/259 (2.3%)	4/40 (10.0%)	299	0.032	4.32 [1.27 to 14.63]
Postoperative Respiratory Failure	4/299 (1.3%)	2/259 (0.8%)	2/40 (5.0%)	299	0.088	6.48 [0.94 to 44.68]
Acute Kidney Injury	20/200 (10.0%)	15/173 (8.7%)	5/27 (18.5%)	200	0.158	2.14 [0.84 to 5.40]
Length of Stay (days)	2.44 ± 2.44	2.37 ± 2.45	2.88 ± 2.36	299	0.219	0.50 [-0.31 to 1.31]
Mortality	6/299 (2.0%)	4/259 (1.5%)	2/40 (5.0%)	299	0.185	3.24 [0.61 to 17.10]

Abbreviations: ASA, American Society of Anesthesiologists Physical Status Classification; BMI, body mass index; CI, confidence interval; RR, relative risk; SST, surgical start time.

### 3.3 Univariate analysis of surgical start times

There was a greater incidence of postoperative blood transfusion in the 15:00–18:43 SST cohort (4/40 vs. 6/259, relative risk 4.32, 95% confidence interval 1.27 to 14.63, p = 0.032). Additionally, there was a greater incidence of weekend operation (including Friday after 15:00) in the 15:00–18:43 SST cohort (17/40 vs. 1/259, relative risk 110.07, 95% confidence interval 15.06 to 804.54, p < 0.001). There was no difference in postoperative respiratory failure, acute kidney injury, hospital length of stay, and mortality between the two SST cohorts (Table 1). Postoperative sepsis (n = 2), venous thromboembolism (n = 0), stroke (n = 0), and myocardial infarction (n = 0) were ultimately excluded from analysis due to a rare event rate (n < 3). The findings of the primary analysis did not differ considerably from the findings of the preliminary analysis which was conducted prior to exclusion by surgeon (S1 Table).

### 3.4 Multivariate analysis of postoperative blood transfusion

The only preoperative or operative characteristics associated with postoperative blood transfusion on univariate testing were SST (4/10 vs. 36/289, relative risk 4.32, 95% confidence interval 1.27 to 14.63, p = 0.032) and mean expired sevoflurane (1.46 ± 0.31 vs. 1.62 ± 0.49, absolute difference 0.17, 95% confidence interval 0.01 to 0.42, p = 0.044) (Table 2). Weekend operation (including Friday after 15:00), primary surgeon, and surgical approach (open versus laparoscopic versus robotic) were not associated with postoperative blood transfusion.

**Table 2:** Univariate comparisons between transfusion cohorts.

Characteristic	No Transfusion n = 289	Transfusion n = 10	n	p-value	Difference or RR [95% CI]
Age (years)	46.63 ± 11.33	50.50 ± 7.50	299	0.224	3.87 [-3.00 to 11.00]
Sex (male)	41/289 (14.2%)	1/10 (10.0%)	299	1.000	0.68 [0.09 to 5.23]
BMI (kg/m^2^)	48.33 ± 10.69	48.42 ± 9.50	299	0.960	0.09 [-5.82 to 6.18]
ASA 2	109/289 (37.7%)	3/10 (30.0%)	299	0.779	
ASA 3	176/289 (60.9%)	7/10 (70.0%)	299	0.779	
ASA 4	4/289 (1.4%)	0/10 (0.0%)	299	0.779	
Diabetes	147/289 (50.9%)	6/10 (60.0%)	299	0.750	1.43 [0.41 to 4.97]
Hypertension	156/289 (54.0%)	7/10 (70.0%)	299	0.356	1.95 [0.51 to 7.38]
Preoperative Hemoglobin (g/dL)	13.23 ± 1.45	12.41 ± 1.29	190	0.161	-0.81 [-1.90 to 0.30]
Preoperative Creatinine (mg/dL)	0.89 ± 0.60	0.83 ± 0.23	194	0.928	-0.06 [-0.10 to 0.10]
15:00–18:43 SST	36/289 (12.5%)	4/10 (40.0%)	299	0.032	4.32 [1.27 to 14.63]
Weekend	17/289 (5.9%)	1/10 (10.0%)	299	0.468	1.73 [0.23 to 12.95]
Surgeon A	144/289 (49.8%)	6/10 (60.0%)	299	0.750	1.49 [0.43 to 5.17]
Surgeon B	145/289 (50.2%)	4/10 (40.0%)	299	0.750	0.67 [0.19 to 2.33]
Bypass—Open	14/289 (4.8%)	1/10 (10.0%)	299	0.441	
Bypass—Laparoscopic	216/289 (74.7%)	8/10 (80.0%)	299	0.441	
Bypass—Robotic	59/289 (20.4%)	1/10 (10.0%)	299	0.441	
Concurrent Procedure	78/289 (27.0%)	4/10 (40.0%)	299	0.470	1.76 [0.51 to 6.09]
Operative Time (min)	171.63 ± 44.04	171.60 ± 47.97	299	0.871	-0.03 [-30.00 to 39.00]
Mean Expired Sevoflurane (%)	1.62 ± 0.49	1.46 ± 0.31	257	0.044	-0.17 [-0.42 to -0.01]
Mean Expired Sevoflurane <1.0%	26/247 (10.5%)	1/10 (10.0%)	257	1.000	0.95 [0.12 to 7.19]
Estimated Blood Loss (mL)	42.54 ± 61.14	41.67 ± 52.04	165	1.000	-0.88 [-50.00 to 75.00]

Abbreviations: ASA, American Society of Anesthesiologists Physical Status Classification; BMI, body mass index; CI, confidence interval; RR, relative risk; SST, surgical start time.

Multivariate analysis demonstrated that after adjusting for covariates, SST was the only independent predictor of postoperative blood transfusion (adjusted odds ratio 4.32, 95% confidence interval 1.06 to 15.96, p = 0.029). The model discrimination was fair (c-statistic = 0.638).

### 3.5 Postoperative blood transfusion audit

Postoperative hemoglobin was missing in one patient from the study sample. This patient was in the early SST cohort and did not receive a blood transfusion. Despite having a greater hemoglobin concentration immediately postoperatively (12.47 ± 1.60 vs. 11.85 ± 1.39 g/dL, p = 0.024), the late SST cohort experienced a greater 48-hour reduction in hemoglobin concentration (1.12 ± 1.42 vs. 0.60 ± 0.99 g/dL, p = 0.028). There was no difference in nadir hemoglobin concentration or discharge hemoglobin concentration between the two SST cohorts. Compared to the non-transfused cohort, the transfused cohort had a lower postoperative hemoglobin concentration (9.61 ± 1.72 vs. 12.01 ± 1.36 g/dL, p = 0.002), lower nadir hemoglobin concentration (6.46 ± 0.53 vs. 11.36 ± 1.62 g/dL, p < 0.001), greater 48 hour reduction in hemoglobin concentration (3.57 ± 1.52 vs. 0.57 ± 0.90 g/dL, p < 0.001), and lower discharge hemoglobin concentration (9.61 ± 2.10 vs. 11.81 ± 1.57 g/dL, p = 0.009) (Table 3).

**Table 3:** Post-hoc audit comparing postoperative hemoglobin by SST cohort and transfusion cohort.

Postoperative Hemoglobin	07:53–14:59 SST n = 258	15:00–18:43 SST n = 40	p-value	No Transfusion n = 288	Transfusion n = 10	p-value
First (g/dL)	11.85 ± 1.39	12.47 ± 1.60	0.024	12.01 ± 1.36	9.61 ± 1.72	0.002
Nadir (g/dL)	11.20 ± 1.72	11.21 ± 2.41	0.967	11.36 ± 1.62	6.46 ± 0.53	<0.001
48 Hour Reduction (g/dL)	0.60 ± 0.99	1.12 ± 1.42	0.028	0.57 ± 0.90	3.57 ± 1.52	<0.001
Discharge (g/dL)	11.70 ± 1.61	11.99 ± 1.78	0.333	11.81 ± 1.57	9.61 ± 2.10	0.009

Abbreviations: SST, surgical start time.

The transfused cohort was comprised of ten patients receiving an average of 3.50 ± 2.12 units of packed red blood cells at a median (interquartile range) of 1.80 days (1.54, 2.03) postoperatively. Of the ten patients, eight (80.0%) were transfused at a hemoglobin concentration < 7.0 g/dL. The remaining two were transfused at hemoglobin concentrations of 7.0 and 7.1 g/dL. Eight (80%) had a 48-hour reduction in hemoglobin exceeding 2.0 g/dL. Estimated blood loss was only documented in three patients (30.0%). Noteworthy postoperative events included surgical exploration and hematoma evacuation in two patients, however events ranged from no apparent surgical complications to massive transfusion protocol and initiation of extra-corporeal membrane oxygenation (Table 4). While it is difficult to determine whether the reason for increased blood transfusion rate in the late SST cohort is mostly surgical, the need for transfusion appeared to be related to the index surgery in seven of ten (70.0%) cases (patients 1, 3, 5–8, and 10), suggesting that surgical cause is the major reason for blood transfusion in patients after gastric bypass surgery.

**Table 4:** Additional characteristics of the postoperative blood transfusion cohort.

Preoperative					Intraoperative			Postoperative						
ID	ASA	BMI	Hb	Start Time	Duration, min	EBL	Concurrent Procedure	Days to Transfuse	Units Transfused	First Hb	Nadir Hb	48-Hr Reduction Hb	Discharge Hb	Other Events
1	3	43.9	14.3	1500	137	0	NA	1.77	2	10.7	6.8	3.9	13.8	Left lower quadrant subcutaneous hematoma developed. Resolved.
2	3	51.2	13.2	1500	214	NA	Chole-cystectomy	1.80	2	11.1	6.9	4.2	10.5	Bloody urine, large volume drain output. No leak.
3	3	44.8	NA	0700	120	NA	Liver biopsy	0.58	4	7.0	5.9	2.3	6.9	Taken to operating room for exploration on POD#2, hematoma evacuated.
4	3	42.2	12.0	0700	209	25	NA	1.90	2	9.4	6.7	2.7	9.7	No evidence of complications.
5	2	48.5	12.2	0700	93	NA	Hiatal hernia repair	1.46	4	7.6	7.0	1.2	12.4	Taken to operating room for exploration on POD#1, hematoma evacuated.
6	3	69.1	10.7	1500	195	100	NA	6.24	9*	11.2	5.5	6.0	8.5	Readmitted for bowel necrosis versus liver failure; massive transfusion and VV-ECMO cannulation on POD#6. Patient expired on POD#7.
7	3	38.5	NA	0700	171	NA	NA	1.81	2	10.9	7.1	4.3	8.3	Left lower quadrant hematoma developed. Resolved.
8	2	51.2	NA	1500	223	NA	Gastric sleeve	8.45	4	8.4	6.3	3.7	8.5	Readmitted for anastomotic leak on POD#9.
9	3	37.5	13.4	0700	131	NA	NA	2.07	3	11.7	6.3	5.4	9.2	Passed blood in stool, in the setting of prophylactic Enoxaparin administration.
10	2	57.3	11.1	0700	223	NA	NA	0.86	3	8.1	6.1	2.0	8.3	Intraoperative incidental liver laceration.

*Patient 6 received 10 units fresh frozen plasma, 3 units platelets, and 3 units cryoprecipitate, in addition to 9 units packed red blood cells. All other patients received only packed red cells. Abbreviations: ASA, American Society of Anesthesiologists Physical Status Classification; BMI, body mass index (kg/m^2^); EBL, estimated blood loss (mL); Hb, hemoglobin (g/dL); NA, not applicable; POD, postoperative day; VV-ECMO, veno-venous extracorporeal membrane oxygenation.

### 3.6 Receiver operating characteristic analysis

**Fig 2:**
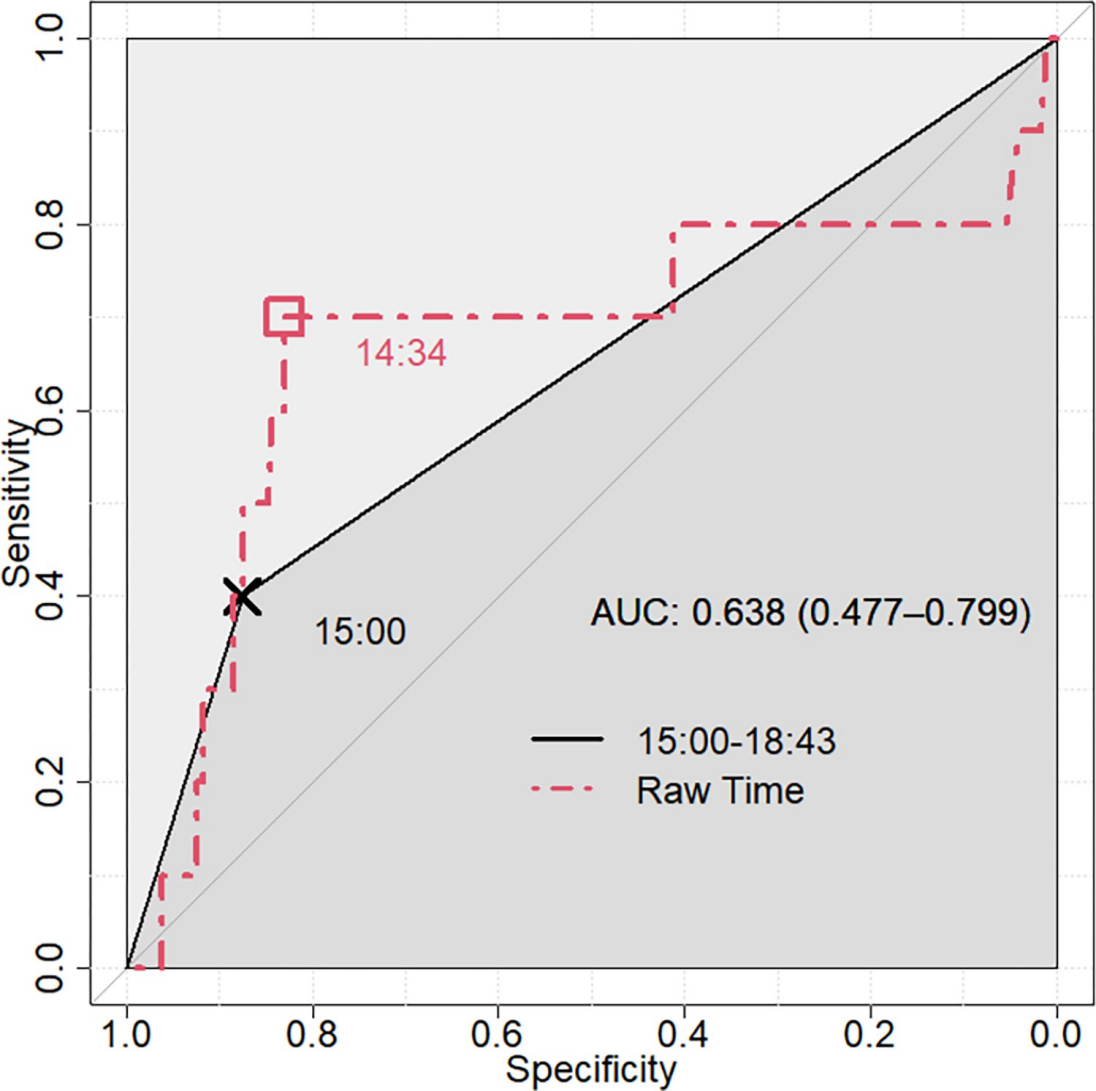
Receiver operating characteristic analysis of postoperative blood transfusion versus case start, performed on both the 15:00 discretization and the raw surgical case start time. Abbreviations: AUC, area under the curve.

On ROC analysis, a 15:00–18:43 SST predicted postoperative blood transfusion with 40.0% sensitivity and 87.5% specificity. A repeated ROC analysis using various SST thresholds demonstrated that accuracy was improved to 70.0% sensitivity and 83.0% specificity at a time threshold of 14:34 (Fig 2). The c-statistic improved from 0.638 to 0.672 under the 14:34 threshold.

## 4. Discussion

This study suggests that a later SST (15:00–18:43) is associated with anemia requiring postoperative blood transfusion. Late SST remained independently predictive of postoperative blood transfusion after adjusting for confounding variables. Despite the arbitrary use of 15:00 as the separation point for the cohorts based on the staff shift changes in our institution, the present study identified that an increased risk of transfusion began at 14:34. Postoperative transfusion was generally appropriate, almost always triggered by a hemoglobin concentration < 7.0 g/dL. The development of anemia was associated with a late SST, which is consistent with the finding of an increased rate of blood transfusion in this cohort.

The present study investigated blood transfusion as the primary outcome for several reasons. First, it is an undesirable, but not uncommon, postoperative event. Blood transfusion has been shown to be associated with surgical site infection and venous thromboembolism in the bariatric surgery population [28, 29]. Bleeding disorders have also been shown to be predictive of postoperative morbidity, prolonged hospital stay, readmission, and return to the operating room [36–39]. If blood transfusion can be mitigated with an earlier SST, this may translate to improved outcomes in patients with predisposition to postoperative anemia. Furthermore, as recently seen during the COVID-19 pandemic, the importance of judicious use of blood products has been further emphasized as blood shortages impacted the healthcare system [30]. Of note, the study demonstrated that postoperative transfusion was not associated with estimated blood loss or preoperative hemoglobin. In fact, intraoperative estimated blood loss was inconsistently documented and perhaps spurious, given that the three patients (of ten transfused) had documented volumes of 0 mL, 25 mL, and 100 mL. Additionally, while some of the transfused patients had developed hematomas that occasionally required surgical evacuation, the cause of blood loss was not always related to the index surgery. Medical and surgical causes of anemia were identified (Table 4), but reasons for the increased incidence after 15:00 remains unclear. Contributing factors may include provider fatigue and staffing changes at multiple phases of care (operating room, 15:00; post-anesthesia care unit, variable; acute care floor, 19:00), as well as patients’ physiologic changes with circadian cycle.

Surgical approach (open versus laparoscopic versus robotic) had no effect on the incidence of postoperative blood transfusion (p = 0.441). While the nuances of selecting the surgical approach are outside the scope of this study, it is worth noting that laparoscopic procedures (with or without the surgical robot) are standard-of-care, and significant differences in postoperative morbidity among the three approaches have not been reliably demonstrated [40]. There is a trend towards increased utilization of the surgical robot, given the theoretical advantages of decreased tissue injury, improved wound healing, and augmented suturing dexterity [41]. The distribution of approaches in the present cohort is as follows: open, 5.0%; laparoscopic, 74.9%; robotic, 20.1%.

Several existing studies have shown worse outcomes with later SST, with reported AORs and hazard ratios comparable to that of this study [6–12]. A noteworthy strength of the present study is that it investigates a truly elective surgical population, thereby minimizing the confounding effects of greater case urgency and patient acuity during late SSTs. Linzey et al. (2020) demonstrated a late SST (17:00–07:00) for neurosurgery was associated with emergent case status (46.3% vs. 7.0%, P<0.0001) and with complications on univariate (22.1% vs. 11.0%, P<0.0001) and multivariate analysis (AOR 1.71, 95% CI 1.03–2.84, p = 0.04). However, this significance was not maintained on the elective procedure subgroup [13]. Many existing studies identify a significant univariate association between SST and postoperative morbidity and mortality. However, significance was not maintained after adjusting for covariates, such as case urgency [14–17]. The present study is strengthened by its univariate comparisons demonstrating no difference in comorbidities or ASA physical status classification between SST cohorts. The c-statistics (0.638 at time 15:00 and 0.672 at time 14:34) demonstrate good but not excellent discrimination, however the limitations in quantifying effect size by c-statistic alone are well described [42]. A more important finding was the determination that SST remained an independent predictor of postoperative blood transfusion on multivariate analysis even with adjustment for confounders via stepwise model selection criteria.

Badiyan et al. (2013) applied ROC analysis to demonstrate improved local control of brain tumor and survival following stereotactic neurologic radiosurgery for brain metastases prior to 11:41 am on a univariate level. However, the significance was lost on multivariate and matched-pairs analysis [15]. The present study is the only additional study to apply ROC analysis to validate its SST strata and assess for improved accuracy. Importantly, Lonze et al. (2010) demonstrated that mixed results may be obtained under different SST discretization schema when comparing mortality and operative time in the transplant surgery population [23].

This study is not without limitations. This is an observational study and as such the study does not offer full control over all possible covariates, despite adjusting for numerous potential confounders during the multivariate analysis. This is a recurring theme in this field, as all existing studies on SST have been limited by this design factor. An ideal study design would involve randomization of patients to different SSTs. While this can be more easily accomplished in the gastric bypass population than in those requiring emergent surgery, the need to maintain efficient hospital operations and the inability to blind surgeons remain noteworthy practical limitations. Missingness of preoperative serum laboratory results is a noteworthy limitation. Acute kidney injury could only be retrospectively assessed in 200 of 299 patients due to missing preoperative serum creatinine concentrations. Understandably, this resulted in a loss of statistical power when assessing this particular outcome. Several measures were taken to account for the relatively small size of this study. The sample size was supported by the *a priori* power analysis, Fisher’s exact test was applied given the anticipated rare event rates, and there were appropriately at least ten incident outcome events per independent variable in the multivariate regression [43–45]. Despite this, the results of this study have yet to be validated in a larger, multi-center cohort.

Provider fatigue and handoff of care are important confounders that may be associated with a late SST and increased morbidity. Thus, it may be a reasonable approach to investigate whether SST is associated with complications in animal models, as the laboratory environment can be used to ensure adequate control of additional confounders and perform mechanistic studies. Additionally, the authors acknowledge that different results may be produced under different SST strata or composite endpoints. Alternate SST discretization was appropriately investigated by ROC analysis. However, composite endpoint construction was avoided due to limitations in this methodology [46, 47]. It should also be noted that studying this association at just one institution eliminated between-facilities variability. However, it may limit the generalizability of our results to institutions with similar resources and practices.

## 5. Conclusions

The present study demonstrates an increased risk of postoperative transfusion with later SST in the gastric bypass population. Earlier SST may decrease the incidence of postoperative blood transfusions in elective cases. Further research is warranted to investigate the morbidity and mortality attributable to late SSTs in elective cases.

## Supporting information

S1 Table Demographic data and univariate comparisons between 07:53–14:59 SST and 15:00–18:43 SST cohorts, prior to exclusion by surgeon. Abbreviations: ASA, American Society of Anesthesiologists Physical Status Classification; BMI, body mass index; CI, confidence interval; RR, relative risk; SST, surgical start time.
(DOCX)
