# Supplementary material for: Late surgical start time is associated with increased blood transfusion following gastric bypass surgery
Source: PLoS One. 2023 Feb 24;18(2):e0282139. doi: 10.1371/journal.pone.0282139 (PMC9956042; doi:10.1371/journal.pone.0282139)
Supplement: S1 Table — Abbreviations: ASA, American Society of Anesthesiologists Physical Status Classification; BMI, body mass index; CI, confidence interval; RR, relative risk; SST, surgical start time. (DOCX) [file pone.0282139.s001.docx]

Supplementary Material

# Supplementary Table 1. Demographic data and univariate comparisons between 07:53-14:59 SST and 15:00-18:43 SST cohorts, prior to exclusion by surgeon.

| **Characteristic** | **Aggregate n=311** | **07:53-14:59 SST n=270** | **15:00-18:43 SST n=41** | **n** | **p-value** | **Difference or RR [95% CI]** |
| --- | --- | --- | --- | --- | --- | --- |
| Age (years) | 46.64 ± 11.25 | 46.74 ± 11.42 | 45.98 ± 10.16 | 311 | 0.662 | -0.76 [-4.23 to 2.71] |
| Sex (male) | 43/311 (13.8%) | 35/270 (13.0%) | 8/41 (19.5%) | 311 | 0.328 | 1.51 [0.75 to 3.01] |
| BMI (kg/m^2^) | 48.39 ± 10.64 | 48.36 ± 10.50 | 48.56 ± 11.67 | 311 | 0.919 | 0.20 [-3.68 to 4.07] |
| ASA 2 | 117/311 (37.6%) | 102/270 (37.8%) | 15/41 (36.6%) | 311 | 0.923 |  |
| ASA 3 | 190/311 (61.1%) | 164/270 (60.7%) | 26/41 (63.4%) | 311 | 0.923 |  |
| ASA 4 | 4/311 (1.3%) | 4/270 (1.5%) | 0/41 (0.0%) | 311 | 0.923 |  |
| Diabetes | 160/311 (51.4%) | 138/270 (51.1%) | 22/41 (53.7%) | 311 | 0.867 | 1.05 [0.77 to 1.43] |
| Hypertension | 171/311 (55.0%) | 144/270 (53.3%) | 27/41 (65.9%) | 311 | 0.177 | 1.23 [0.96 to 1.58] |
| Preoperative Hemoglobin (g/dL) | 13.20 ± 1.43 | 13.16 ± 1.40 | 13.44 ± 1.57 | 200 | 0.377 | 0.28 [-0.36 to 0.92] |
| Preoperative Creatinine (mg/dL) | 0.88 ± 0.58 | 0.88 ± 0.61 | 0.86 ± 0.29 | 204 | 0.724 | -0.03 [-0.17 to 0.12] |
| 15:00-18:43 SST | 41/311 (13.2%) | - | - | 311 | - | - |
| Weekend | 18/311 (5.8%) | 1/270 (0.4%) | 17/41 (41.5%) | 311 | <0.001 | 111.95 [15.31 to 818.86] |
| Surgeon A | 150/311 (48.2%) | 129/270 (47.8%) | 21/41 (51.2%) | 311 | 0.385 |  |
| Surgeon B | 149/311 (47.9%) | 130/270 (48.1%) | 19/41 (46.3%) | 311 | 0.385 |  |
| Surgeon C | 6/311 (1.9%) | 6/270 (2.2%) | 0/41 (0.0%) | 311 | 0.385 |  |
| Surgeon D | 3/311 (1.0%) | 3/270 (1.1%) | 0/41 (0.0%) | 311 | 0.385 |  |
| Surgeon E | 2/311 (0.6%) | 2/270 (0.7%) | 0/41 (0.0%) | 311 | 0.385 |  |
| Surgeon F | 1/311 (0.3%) | 0/270 (0.0%) | 1/41 (2.4%) | 311 | 0.385 |  |
| Bypass - Open | 15/311 (4.8%) | 12/270 (4.4%) | 3/41 (7.3%) | 311 | 0.202 |  |
| Bypass - Laparoscopic | 236/311 (75.9%) | 209/270 (77.4%) | 27/41 (65.9%) | 311 | 0.202 |  |
| Bypass - Robotic | 60/311 (19.3%) | 49/270 (18.1%) | 11/41 (26.8%) | 311 | 0.202 |  |
| Concurrent Procedure | 86/311 (27.7%) | 77/270 (28.5%) | 9/41 (22.0%) | 311 | 0.456 | 0.77 [0.42 to 1.41] |
| Operative Time (min) | 172.43 ± 44.76 | 171.60 ± 45.48 | 177.88 ± 39.78 | 311 | 0.360 | 6.28 [-7.34 to 19.90] |
| Mean Expired Sevoflurane (%) | 1.61 ± 0.49 | 1.61 ± 0.50 | 1.65 ± 0.43 | 267 | 0.575 | 0.04 [-0.11 to 0.20] |
| Mean Expired Sevoflurane <1.0% | 28/267 (10.5%) | 25/229 (10.9%) | 3/38 (7.9%) | 267 | 0.777 | 0.72 [0.23 to 2.28] |
| Estimated Blood Loss (mL) | 42.18 ± 59.76 | 41.49 ± 60.59 | 48.53 ± 52.76 | 173 | 0.612 | 7.04 [-21.43 to 35.51] |
| Postoperative Blood Transfusion | 10/311 (3.2%) | 6/270 (2.2%) | 4/41 (9.8%) | 311 | 0.030 | 4.39 [1.29 to 14.90] |
| Postoperative Respiratory Failure | 4/311 (1.3%) | 2/270 (0.7%) | 2/41 (4.9%) | 311 | 0.086 | 6.59 [0.95 to 45.47] |
| Acute Kidney Injury | 21/210 (10.0%) | 16/182 (8.8%) | 5/28 (17.9%) | 210 | 0.169 | 2.03 [0.81 to 5.11] |
| Length of Stay (days) | 2.44 ± 2.41 | 2.37 ± 2.42 | 2.88 ± 2.33 | 311 | 0.204 | 0.50 [-0.28 to 1.29] |
| Mortality | 6/311 (1.9%) | 4/270 (1.5%) | 2/41 (4.9%) | 311 | 0.180 | 3.29 [0.62 to 17.41] |

Abbreviations: ASA, American Society of Anesthesiologists Physical Status Classification; BMI, body mass index; CI, confidence interval; RR, relative risk; SST, surgical start time.
